# Cortical Mechanisms of Tongue Sensorimotor Functions in Humans: A Review of the Magnetoencephalography Approach

**DOI:** 10.3389/fnhum.2017.00134

**Published:** 2017-03-28

**Authors:** Hitoshi Maezawa

**Affiliations:** Department of Oral Physiology, Graduate School of Dental Medicine, Hokkaido UniversitySapporo, Japan

**Keywords:** cortico-muscular coherence, event-related desynchronization/synchronization, hypoglossal nerve, movement-related cortical fields, somatosensory evoked fields, trigeminal nerve

## Abstract

The tongue plays important roles in a variety of critical human oral functions, including speech production, swallowing, mastication and respiration. These sophisticated tongue movements are in part finely regulated by cortical entrainment. Many studies have examined sensorimotor processing in the limbs using magnetoencephalography (MEG), which has high spatiotemporal resolution. Such studies have employed multiple methods of analysis, including somatosensory evoked fields (SEFs), movement-related cortical fields (MRCFs), event-related desynchronization/synchronization (ERD/ERS) associated with somatosensory stimulation or movement and cortico-muscular coherence (CMC) during sustained movement. However, the cortical mechanisms underlying the sensorimotor functions of the tongue remain unclear, as contamination artifacts induced by stimulation and/or muscle activity within the orofacial region complicates MEG analysis in the oral region. Recently, several studies have obtained MEG recordings from the tongue region using improved stimulation methods and movement tasks. In the present review, we provide a detailed overview of tongue sensorimotor processing in humans, based on the findings of recent MEG studies. In addition, we review the clinical applications of MEG for sensory disturbances of the tongue caused by damage to the lingual nerve. Increased knowledge of the physiological and pathophysiological mechanisms underlying tongue sensorimotor processing may improve our understanding of the cortical entrainment of human oral functions.

## Introduction

Non-invasive electromagnetic imaging techniques such as electroencephalography (EEG) and magnetoencephalography (MEG) are powerful tools for elucidating cortical activity with high temporal resolution. While the spatial resolution of EEG is limited because of the low current conductivity of the skull, MEG offers significantly higher spatial resolution, as magnetic fields are less distorted by tissues of the skull and scalp than electric fields. However, MEG does have some disadvantages. Unlike EEG—which is sensitive to both tangential and radial dipoles—MEG is insensitive to current dipoles that are radial to the skull, since radial dipoles do not contribute to the outer magnetic field detected by the coil. However, one previous study reported that MEG may allow for the visualization of activity from gyral sources with predominantly radial orientation, with the exception of thin strips at the crests of gyri (Hillebrand and Barnes, 2002). In addition, MEG is insensitive to dipoles in deep sources, as magnetic fields rapidly decrease with increasing depth.

Despite its disadvantages, MEG is especially useful for detecting activation of the sensorimotor cortex, which originates from the wall of the central sulcus (Brodmann areas [BA] 3b and 4), because of its ability to detect dipoles tangential to the head surface. Indeed, many MEG studies have investigated the cortical processes related to sensorimotor functions of the limbs in humans. Such studies have employed multiple analysis methods, including somatosensory evoked fields (SEFs), movement-related cortical fields (MRCFs), and frequency analyses using event-related desynchronization/event-related synchronization (ERD/ERS) and cortico-muscular coherence (CMC; Hari and Salmelin, 1997, 2012; Hari and Salenius, 1999; Kakigi et al., 2000; Shibasaki and Hallett, 2006; Shibasaki, 2012; Cheyne, 2013).

Oral functions involving voluntary movements (e.g., speech) are mainly regulated by cortical control. However, research has revealed that some aspects of oral functions involving automatic movements (e.g., chewing, swallowing, respiration) are also under voluntary control (Martin and Sessle, 1993; Hamdy et al., 1999a,b; Martin-Harris, 2006; Matsuo and Palmer, 2009). Such findings suggest that the cortex plays critical roles in tongue movement, particularly when executing fine oral functions. The somatosensory sensations and movements of the tongue are exerted largely by the crossed and uncrossed fiber tracts that run through the ascending and descending pathways of both hemispheres. Several previous studies have indicated that the oral region is represented in the ipsilateral primary somatosensory cortex (S1; Ogawa et al., 1989; Manger et al., 1995, 1996; Jain et al., 2001) and primary motor cortex (M1; Martin et al., 1997) in nonhuman primates. There is also evidence of ipsilateral oral representation in S1 and M1 in humans. Penfield and Rasmussen (1950) reported that unilateral, direct cortical stimulation of the S1 and M1 cortices induced bilateral tongue sensation and movement in humans, respectively.

**Figure 1:**
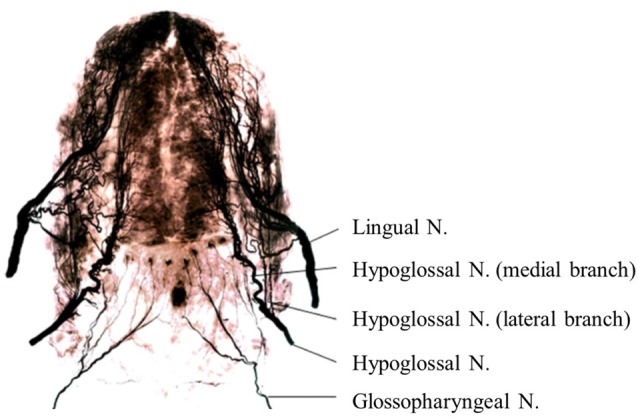
**Nerve maps of the whole tongue in the human adult using Sihler’s stain.** Note that the hypoglossal nerve (Hypoglossal N.) and its branches are located between the lingual nerve (Lingual N.) and glossopharyngeal nerve (Glossopharyngeal N.) in the posterior tongue. Images modified with permission from Mu and Sanders (2010).

Anatomically, the sensorimotor functions of the human tongue are controlled by multiple cranial nerves (CNs; Sawczuk and Mosier, 2001; Zur et al., 2004; Mu and Sanders, 2010). Somatosensory innervation of the tongue is supplied by the lingual nerve (mandibular branch of the trigeminal nerve (CN V); anterior two thirds of the tongue), glossopharyngeal nerve (CN IX; posterior one-third of the tongue; Figure 1), and vagus nerve (posterior region of the tongue root; CN X). Deep sensations in the tongue muscle are transmitted by the hypoglossal nerve (CN XII). Motor innervation of the tongue is supplied by the hypoglossal nerve (CN XII), except for the platoglossus muscle, which is innervated by the vagus nerve (CN X).

As the tongue region in humans has fine somatosensory sensation and can perform sophisticated movements, the area of the primary sensorimotor cortex that represents the tongue occupies a wide distribution relative to its actual size in the body (Penfield and Rasmussen, 1950). However, relatively few studies have examined the cortical mechanisms related to sensorimotor functions of the tongue using MEG, as it is difficult to measure MEG signals during tongue stimulation and movement without artifact contamination because of the short distance between the tongue and brain. However, some MEG studies have successfully recorded sensorimotor functions of the tongue using improved methods of tongue stimulation and movement tasks. In the present review, we provide a detailed overview of tongue sensorimotor processing in humans, based on the findings of MEG studies that have utilized multiple analysis methods, such as SEFs, MRCFs, ERD/ERS and CMC. In addition, we review the clinical applications of SEFs for patients with sensory disturbances of the tongue caused by damage to the lingual nerve.

## The Physiology of Tongue Sensorimotor Processing

### Evoked Fields

Following the development of the initial MEG-based recordings of evoked responses to somatosensory stimulation (Brenner et al., 1978; Kaufman et al., 1981; Hari et al., 1983b) and voluntary movement (Deecke et al., 1982; Hari et al., 1983a; Weinberg et al., 1983), SEFs and MRCFs have been utilized to examine sensorimotor processing in the upper and lower limbs in both clinical and research-based investigations. In this section, we review sensorimotor processing of the tongue region as visualized using SEFs and MRCFs.

#### Somatosensory Evoked Fields (SEFs)

Since the SEFs for electrical tongue stimulation were first reported in the early 1990s (Karhu et al., 1991), several tongue SEF studies have been conducted using electrical (Nakahara et al., 2004; Maezawa et al., 2008; Sakamoto et al., 2008a) and mechanical stimulation (Nakamura et al., 1998; Yamashita et al., 1999; Disbrow et al., 2003; Tamura et al., 2008).

**Figure 2:**
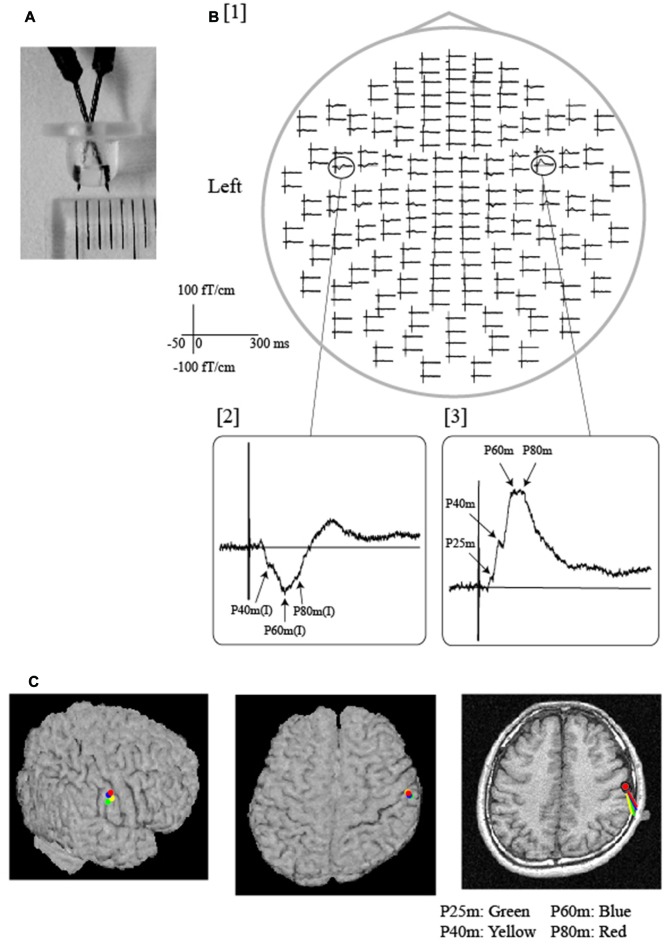
**Somatosensory evoked fields (SEFs) following electrical tongue stimulation using pin electrodes in a representative participant. (A)** A pair of pin electrodes was used to safely stimulate the tongue at a low intensity. The tip of the electrode measured 3 mm across. **(B)** [1] The whole-head SEF waveforms exhibited obvious responses in both hemispheres. The time window of each waveform was set from −50 ms to 300 ms with respect to stimulus onset. As shown in the expanded waveforms [2, 3], middle-latency components were detected over the contralateral and ipsilateral hemispheres. Note that artifacts induced by electrical stimulation were effectively reduced by the low stimulus intensity produced by the pin electrodes. **(C)** Equivalent current dipoles (ECDs) of each component (P25 m, P40 m, P60 m, P80 m) were superimposed on each participant’s magnetic resonance and surface rendering images. All ECDs were positioned over the same area on the posterior bank of the central sulcus, which is thought to represent the location of the tongue region of the primary somatosensory cortex (S1). The direction of the line represents the negative pole of the ECDs. All ECDs were directed posteriorly in a similar manner. Images modified with permission from Maezawa et al. (2008).

The initial component of tongue SEFs was observed over the bilateral hemispheres at 19 ms (Sakamoto et al., 2008a) and 14 ms (Tamura et al., 2008), respectively, with an anterior current orientation. The middle-latency component of tongue SEFs was also identified over both hemispheres at a peak latency ranging from 25 ms to 80 ms, with a posterior current orientation (Figure 2; Karhu et al., 1991; Nakamura et al., 1998; Yamashita et al., 1999; Disbrow et al., 2003; Nakahara et al., 2004; Maezawa et al., 2008, 2014a). Further investigation revealed that the initial and middle-latency components of tongue SEFs derived from the bilateral S1—specifically, the posterior bank of the central sulcus—although contralateral dominance was observed (Tamura et al., 2008). This finding may have an anatomical basis, as the unilateral lingual nerve that innervates the anterior portion of the tongue projects to the bilateral BA3b via the trigeminothalamic tract, with contralateral dominance. While the middle-latency component of tongue SEFs is relatively easy to record because of its high amplitude, the amplitude of the initial component is substantially lower, rendering recording somewhat difficult. As such, the middle-latency component is often used as a reliable parameter in clinical situations (Figure 2; Maezawa et al., 2008, 2011). The different current orientations of the initial and middle-latency components suggest that different cortical mechanisms may underlie these components, although further studies are required to elucidate the precise mechanisms underlying each tongue SEF component (Sakamoto et al., 2010).

Additional MEG studies have differentiated between responses generated in the tongue area of S1 and those generated in the secondary somatosensory cortex (S2), based on the latency of the SEFs and the direction of the source current (Karhu et al., 1991; Disbrow et al., 2003; Sakamoto et al., 2008b). The response from the tongue area of S2 was detected at 80–110 ms after electrical stimulation, and the equivalent current dipoles (ECDs) of S2 were directed superiorly (Sakamoto et al., 2008b). Such studies have indicated that the tongue area of S2 is located in the upper bank of the sylvian fissure, close to the hand area of S2 and significantly more anterior than the foot area of S2, suggesting that the tongue area of S2 occupies a small region with roughly somatotopic organization (Sakamoto et al., 2008b).

#### Movement-Related Cortical Fields (MRCFs)

##### MRCFs associated with tongue protrusions

**Figure 3:**
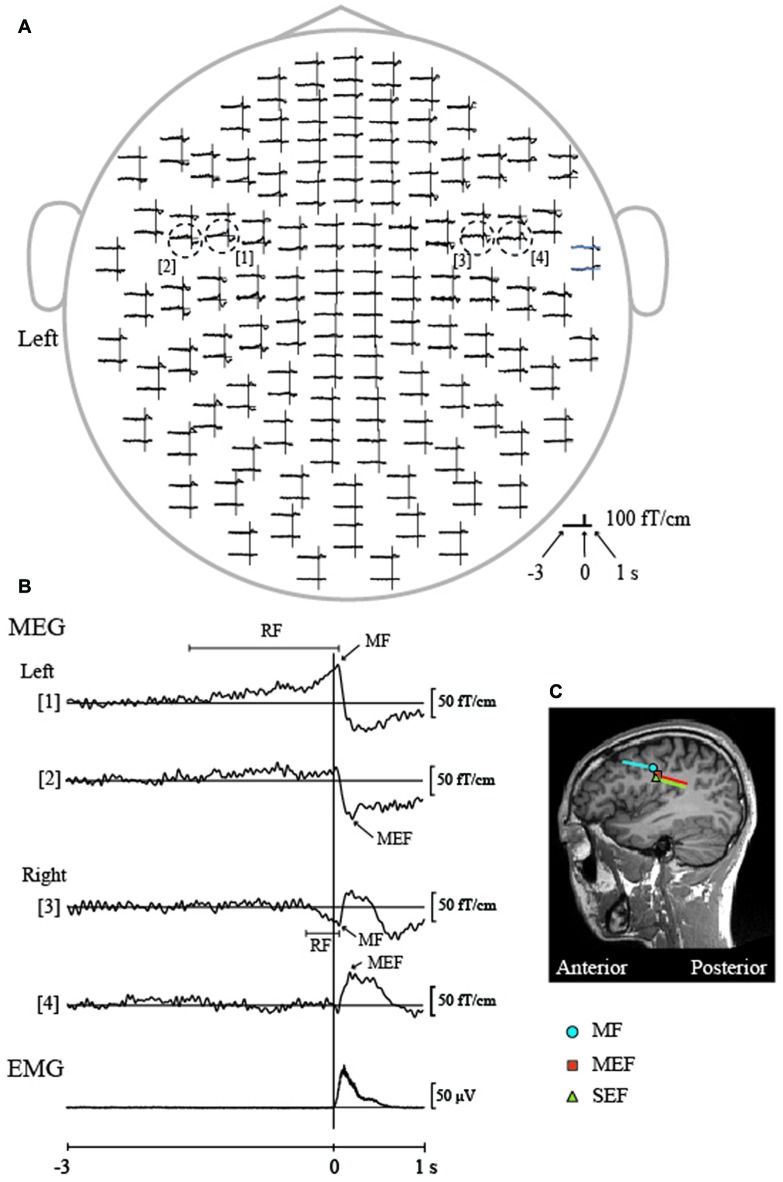
**Movement-related cortical fields (MRCFs) associated with self-paced tongue protrusion in a representative participant. (A)** The whole-head magnetic waveforms of the MRCFs associated with tongue protrusion in one participant. The vertical lines represent the onset of tongue electromyography (EMG). The time window of each waveform was set from −3 s to 1 s with respect to EMG onset. MRCFs were detected over the sensorimotor areas in both hemispheres. The dotted circles indicate the waveforms with the maximum amplitude for each MRCF component (motor fields, MFs, [1, 3] and motor-evoked fields, MEFs, [2, 4]) in each hemisphere. **(B)** Enlarged magnetic waveforms from within the dashed circle in **(A)** and the rectified and averaged EMG signal of the tongue. A slow pre-movement component of the readiness fields (RFs) was observed bilaterally prior to movement onset ([1], left hemisphere; [3], right hemisphere) and culminated in the MFs just after movement onset. MEFs appeared bilaterally after movement onset ([2], left hemisphere; [4], right hemisphere). **(C)** Locations of the ECDs over the left hemisphere for the MF and MEF of the MRCF associated with tongue protrusion, and SEF by tongue stimulation using pin electrodes. The ECDs of the MF were positioned on the anterior bank of the central sulcus, which is thought to represent the primary motor cortex (M1). The ECDs of the MEF and SEF were positioned on the posterior bank of the central sulcus, which is thought to represent the S1. The direction of the line represents the negative pole of the ECDs. The MF ECDs were directed anteriorly, while the MEF and SEF ECDs were directed posteriorly. MEG, Magnetoencephalography. Images modified with permission from Maezawa et al. (2016b).

Cheyne et al. (1991) first reported that MRCFs over the left hemisphere were associated with repetitive tongue protrusions in a single participant using a 7-channel MEG system. A subsequent study by Nakasato et al. (2001) demonstrated whole-head MRCFs for tongue protrusion in five healthy volunteers using a trigger signal, which was used to sense when the tip of the tongue had reached the anterior region of the palate. The authors successfully determined that the ECDs of the MRCFs were located in the tongue region of M1. However, because of inter-individual variability in the time delay between the trigger signal and the onset of tongue movement, the temporal resolution was insufficient, and the MRCF components that occurred before and after tongue movement could not be separated. Recently, we successfully demonstrated bilateral MRCFs both before and after voluntary self-paced tongue movement using a trigger signal based on tongue electromyogram (EMG) data. In accordance with the findings of finger MRCF studies, we detected three components in response to tongue movement: readiness fields (RFs), motor fields (MFs), and movement-evoked fields (MEFs; Figure 3; Maezawa et al., 2016b). We observed slow, bilateral RF components prior to the onset of movement, and these components peaked in MFs near movement onset. These findings may be explained by the anatomical connections between the brain and tongue, as the unilateral hypoglossal nerve (CN XII) from each hemisphere projects to both sides of the tongue through the hypoglossal nuclei via the corticobulbar tract. Since the MF component appeared after the pre-movement RF component and originated from the bilateral M1, this suggests that the bilateral M1 is involved in the preparation and execution of tongue movements. In contrast, the MF component, which appeared after movement onset and originated from the bilateral S1, may reflect proprioceptive feedback from the tongue during tongue movement.

Nagamine et al. (1996) utilized simultaneous MEG and EEG recording during a unilateral finger movement task, observing differential scalp waveform distributions between MRCFs and movement-related cortical potentials (MRCPs). The authors also reported that the slow, pre-movement RF component detected by MEG (the late Bereitschaftspotential [BP] in EEG) appears much later than that detected by EEG (early BP) and occurs in the contralateral M1. These findings may be associated with the inherent difficulty in detecting dipoles with sources that are directed radially to the surface of the skull when using MEG, whereas EEG can record dipoles with both radially- and tangentially-directed sources. In contrast, the RF component can be detected by both MEG and EEG because of its tangential orientation. However, the early BP can only be detected by EEG owing to its bilateral radial orientation in the lateral premotor area (Shibasaki and Hallett, 2006). Indeed, in a previous study of seven patients with epilepsy, Ikeda et al. (1995) performed invasive EEG recordings, reporting that the early BP began to appear over the lesioned hemisphere from 0.9 s to 1.6 s. However, future studies should compare each MRCF component to MRCPs associated with self-paced tongue protrusion, to more fully elucidate the precise cortical mechanisms underlying their generation.

##### MRCFs associated with speech and swallowing

Based on the findings of previous studies that have investigated BP prior to speech onset (McAdam and Whitaker, 1971), several studies have observed that specific patterns of movement-related cortical activation are associated with speech production using EEG (Deecke et al., 1986; Wohlert, 1993), invasive EEG (Ikeda et al., 1995), and MEG (Gunji et al., 2000). Previous studies have reported that the topographic features of MRCPs/MRCFs associated with the vocalizations of single words were almost identical to those of MRCPs/MRCFs associated with simple tongue protrusions (Wohlert, 1993; Ikeda et al., 1995; Gunji et al., 2000). However, these results are in contrast with previous indications that the late BP (final 100 ms to speech onset) is significantly lateralized over the left hemisphere when analyzed using EEG (Deecke et al., 1986). Although the reason underlying this contrast remains uncertain, the differential results may be associated with differences among the vocalization task paradigms of the studies. MRCPs/MRCFs associated with vocalization are influenced by repetition as well as the number of syllables of the vocalization. In the vocalization task of Ikeda et al. (1995), participants were asked to spontaneously utter single or various words, and to avoid unnecessary movement in the tongue and orofacial region during recording. In Gunji et al. (2000), participants were instructed to repeatedly utter a specific vowel (“u”) at a self-paced interval of approximately 5 s. This vowel was selected to minimize EMG activity from oral and tongue muscles required for pronunciation. Participants were also instructed to keep the tongue on the floor of the mouth to reduce artifacts associated with tongue movement. Wohlert (1993) utilized either simple word vocalization (e.g., “pool”) or non-speech oral movement tasks (e.g., lip pursing and lip rounding). In yet another study (Deecke et al., 1986), subjects were asked to utter words beginning with the letter “p” at an irregular interval between 4 s and 12 s. Further studies are required to determine whether the different parameters of these vocalization tasks account for differences in the hemispheric dominance of MRCPs/MRCFs across studies.

In addition, some researchers have utilized EEG to analyze MCRPs during swallowing (Huckabee et al., 2003; Satow et al., 2004). Satow et al. (2004) noted that the post-movement potential was significantly larger during simple tongue protrusion than during swallowing, suggesting that the cortex may not be involved in the post-movement processing of swallowing signals.

### Oscillatory Activity

Previous EEG studies in the human sensorimotor cortex have documented brain rhythm modulations associated with somatosensory stimulation (Jasper and Andrews, 1938), as well as those associated with voluntary, passive and imagined movements (Jasper and Penfield, 1949; Gastaut et al., 1952; Chatrian et al., 1959). Several MEG studies have also focused on the brain rhythms associated with sensorimotor functions in the limbs and, more recently, in the tongue. Although the precise mechanisms underlying such brain rhythms remain to be determined (Engel and Fries, 2010; van Wijk et al., 2012), this section presents the current knowledge and theories regarding the mechanisms of tongue sensorimotor processing, based on the findings of studies that utilized the following frequency analyses: (1) ERD/ERS associated with tongue stimulation and movement; and (2) CMC during sustained tongue protrusion.

#### Event-Related Desynchronization/Event-Related Synchronization (ERD/ERS)

##### ERD/ERS associated with movements of the upper limb

Pfurtscheller and colleagues investigated changes in the oscillatory power of specific frequency bands (e.g., alpha (α) and beta (β) bands) associated with somatosensory stimulation and voluntary movement. The authors reported that event- or task-induced decreases in ERD represented increased cortical activation of the sensorimotor cortex, while similar increases in ERS reflected a recovery of decreased cortical activation (Pfurtscheller and Aranibar, 1977; Pfurtscheller and Lopes da Silva, 1999). Alternatively, several recent reports have suggested that ERS is a signature of active stabilization processes in the sensorimotor cortex (Caetano et al., 2007), whereby external input and activation by new movements are blocked (Gilbertson et al., 2005). More recently, oscillatory activity in the β frequency band has been associated with the maintenance of the current sensorimotor state (Engel and Fries, 2010). Little and Brown (2014) further suggested that β-band activity is causally and quantitatively critical for motor impairment in Parkinson’s disease.

**Figure 4:**
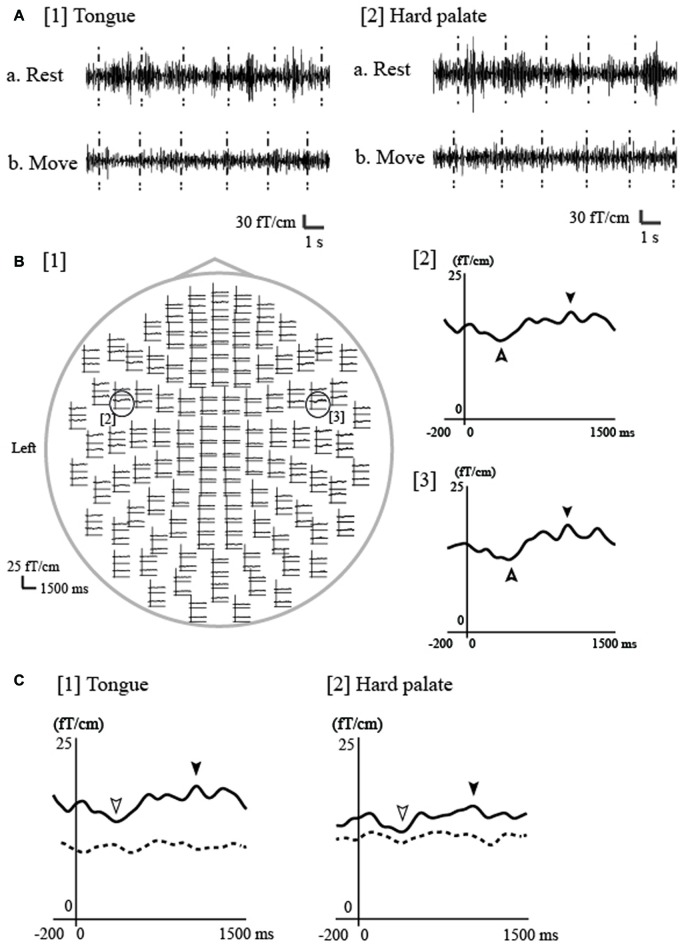
**Event-related desynchronization/event-related synchronization (ERD/ERS) associated with electrical tongue stimulation using pin electrodes in a representative participant. (A)** The continuous magnetic signal was bandpass filtered at 18–23 Hz from a channel in the contralateral (left) hemisphere during electrical stimulation of the tongue and hard palate on the right side. The timing of stimulation is marked with vertical dashed lines. Bursts of 20 Hz oscillation appeared for both the tongue ([1]a) and hard palate ([2]a) during rest, but disappeared during repetitive tongue movement for both regions ([1]b, [2]b). Rest, during the rest period; Move, during the repetitive tongue movement. **(B)** [1] The whole-head waveforms of ERD/ERS at 20 Hz induced by tongue stimulation of the right side in one participant. The ERD/ERS were evaluated using the temporal spectral evolution (TSE) method reported by Salmelin and Hari (1994a) and Hari and Salenius (1999). Using the TSE method, the continuous magnetic signals were filtered at 18–23 Hz and rectified. The rectified MEG signals were averaged to the stimulation and smoothed with a 10-Hz low-pass filter. The top view shows obvious responses in both hemispheres. The time window of each waveform was set from −200 ms to 1500 ms with respect to stimulus onset. ERS and ERD were identified in the contralateral ([2]) and ipsilateral ([3]) hemispheres during rest (indicated by filled arrowheads [ERS] and outlined arrowheads [ERD]).** (C)** ERD/ERS at 20-Hz during the rest and move conditions for the tongue and hard palate over the contralateral hemisphere in one participant. The waveforms obtained during rest and tongue movement are indicated by the solid and dashed lines, respectively. Each waveform was obtained from 200 ms before stimulation onset to 1500 ms after stimulation onset. ERS was detected for both regions (tongue and hard palate) during rest, but was obviously suppressed during tongue movement. Images modified with permission from Maezawa et al. (2016c).

Most MEG studies on electrical stimulation-induced ERD/ERS have focused on the upper limbs (Salmelin and Hari, 1994a,b; Salenius et al., 1997). However, in these studies, the stimulus intensity was above the motor threshold, which may have led to proprioceptive feedback effects from the muscles or joints. Thus, whether the ERD/ERS was induced by “pure” cutaneous stimulation without the effects of proprioception remained unclear. My laboratory recently reported that ERD/ERS at 20 Hz in the β frequency band (β-ERD/ERS) was observed in the sensorimotor cortex bilaterally following tongue and hard palate stimulation, respectively, at rest (Figure 4; Maezawa et al., 2016c) using temporal spectral evolution (TSE) analysis (Salmelin and Hari, 1994a; Hari and Salenius, 1999). In the TSE analysis, the continuous magnetic signals were filtered at 18–23 Hz and rectified, following which the rectified MEG signals were averaged to the stimulation. Cutaneous stimulation can be applied to the hard palate without the effects of proprioception, as this region lacks muscle and joint receptors. Therefore, the detection of β-ERD/ERS for the hard palate suggests that these ERD/ERS signals were induced by pure cutaneous stimulation. In contrast, when stimulating the tongue, the effects of proprioception from the tongue cannot be excluded, as this region is rich in muscle spindles. Furthermore, the β-ERD/ERS signals for both the hard palate and tongue were inhibited during tongue movement, suggesting that—although the stimulation and movement regions differed—movement may have modulated the stimulus-induced functional state of the sensorimotor cortex.

Recent reports using transcranial alternating-current stimulation have noted that enhanced cortical activity at 20 Hz impairs the performance of new movements in the hands, suggesting that cortical activity in the β frequency band may play a critical role in motor behaviors (Pogosyan et al., 2009; Joundi et al., 2012). Given these results, sensorimotor functions of the oral area including the tongue may be finely regulated by oscillatory activity in the β frequency band.

ERD/ERS exhibit temporospatial patterns that differ from those of MRCPs/MRCFs associated with finger movements. In the α frequency band, bilateral ERD begins as early as 2–3 s prior to voluntary movements. In the β frequency band, bilateral ERD appears later and exhibits contralateral dominance (Nagamine et al., 1996; Shibasaki and Hallett, 2006). Following the termination of movement, the slow ERD suddenly terminates as well. However, strong ERS begins in the β frequency band 400–500 ms after the onset of movement (Nagamine et al., 1996). The different temporospatial patterns between ERD/ERS and MRCPs/MRCFs during finger movements suggest that ERD/ERS and MRCPs/MRCFs reflect different underlying generators and functional roles for the preparation and control of finger movement. Rektor et al. (2006) reported that BP is closely related to the direct execution of movement, while ERD/ERS more broadly reflects the cognitive aspects of motor execution (e.g., memory, time interval estimation, executive functions and attention).

##### ERD/ERS associated with simple tongue protrusions, speech and swallowing

Several previous studies have investigated ERD/ERS associated with tongue movement using invasive EEG recordings (Crone et al., 1998; Miller et al., 2007). Crone et al. (1998) investigated the generation of ERD at 15–25 Hz, revealing a somatotopic representation among the tongue, arm and foot. However, since little is known regarding the cortical mechanisms underlying tongue movement-related ERD/ERS measured via non-invasive MEG, more information can be obtained by comparing the spatiotemporal patterns of ERD/ERS to those of MRCFs.

Salmelin and Sams (2002) investigated ERD/ERS associated with tongue and lip movement during verbal and non-verbal tasks at 20 Hz using MEG. The authors observed ERD/ERS over the hand and oral regions of the bilateral sensorimotor cortex in both types of task. Although no significant differences in ERD of the oral regions were observed between the verbal and non-verbal tasks, ERS was significantly lateralized over the left hemisphere during verbal tasks only. Moreover, ERD of the hand regions was detected during the non-verbal task but was diminished during the verbal task. The authors concluded that the cortical processing differences between the oral and hand areas were more clearly distinguished for the verbal movement task than they were for the non-verbal movement task. Moreover, Gunji et al. (2007) reported that the α-ERD observed during singing was more remarkable in the right sensorimotor cortex relative to that for other vocalization conditions (speaking and humming), suggesting the hemispheric dominance of the regions of the sensorimotor cortex associated with melody generation and motor control during singing.

Swallowing is mainly controlled by two central regions: the brainstem (Jean, 1984) and cerebral cortex (Martin and Sessle, 1993; Hamdy et al., 1999a,b). Dziewas et al. (2003) reported that β-ERD was strongly dominant in the left sensorimotor cortex during volitional water swallowing, less strongly dominant during reflexive water swallowing, and non-dominant during simple tongue movement using MEG combined with synthetic aperture magnetometry analysis. These results suggest that hemispheric lateralization related to swallowing may be influenced by the complexity of movement execution. In a study involving MEG combined with synthetic aperture magnetometry, Furlong et al. (2004) reported that changes in oscillatory power occurred in similar regions during swallowing and simple tongue movements. In another study, researchers obtained extracellular recordings from single neurons following intracortical microstimulation in monkeys, revealing that many neurons in the region of the tongue M1 associated with swallowing have orofacial mechanoreceptive fields (Martin et al., 1997). These findings suggest that afferent sensory feedback from the tongue may strongly contribute to the regulation of swallowing. Given that ERD/ERS associated with movement is less affected by artifacts caused by oral movements than phase-locked evoked responses (e.g., MRCFs; Salmelin, 2007), future studies should investigate ERD/ERS associated with tongue movements executed during oral functions such as speech production and swallowing.

#### Cortico-Muscular Coherence (CMC)

**Figure 5:**
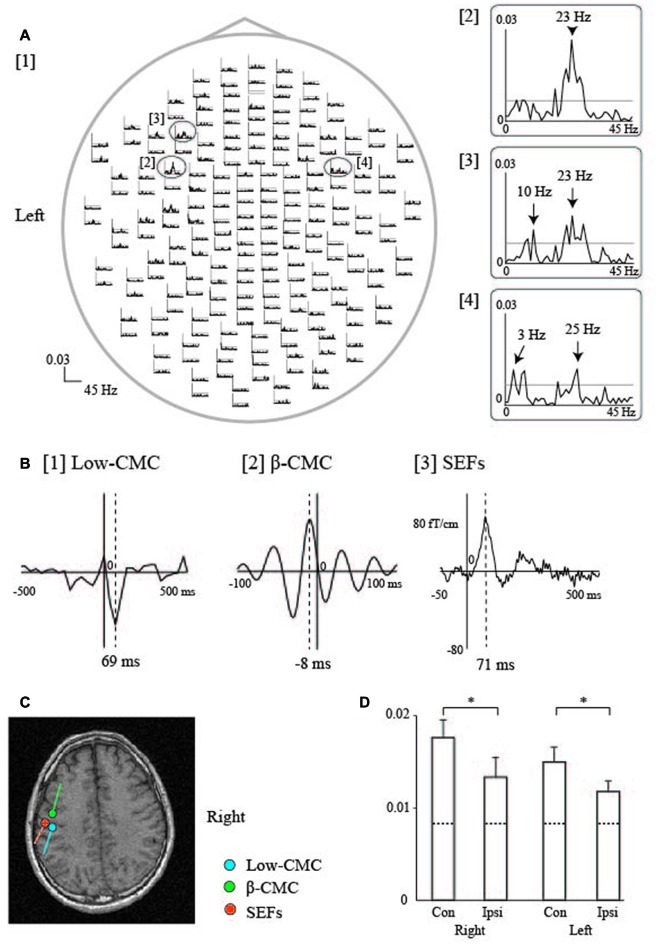
**Cortico-muscular coherence (CMC) during sustained tongue protrusion in a representative participant. (A)** Waveforms of tongue CMC during sustained tongue protrusion. Welch’s method was used to calculate β-CMC, with a frequency resolution of 1 Hz and non-overlapping samples. [1] The whole-head CMC waveforms display obvious peaks in both hemispheres with contralateral (left) dominance. Each trace shows data from 0 Hz to 45 Hz. The 99% significance level is indicated by the horizontal gray line in each column. [2–4] Enlarged traces from the ovals in [1] represent the two peaks observed in the maximum amplitude channels over the contralateral [2, 3] and ipsilateral [4] hemispheres. The β-CMC peaks were detected at 23 Hz over the contralateral hemisphere [2] and at 25 Hz over the ipsilateral hemisphere [4]. CMC at the low frequency band (low-CMC) peaked at 10 Hz [3] over the contralateral hemisphere and at 3 Hz [4] over the ipsilateral hemisphere. **(B)** Cross-correlogram of the β-CMC, low-CMC, and SEF waveforms over the contralateral (left) hemisphere for the right side of the tongue in one participant. Welch’s method was used to calculate the low-CMC, with a frequency resolution of 0.5 Hz and half-overlapping samples. [1] Cross-correlogram of low-CMC. The time window was set from −500 ms to 500 ms with respect to electromyography (EMG) onset. The time of zero lag is indicated by the vertical line. The largest MEG signal peak was detected at 69 ms after EMG onset. [2] Cross-correlogram of β-CMC. The time window was set from −100 ms to 100 ms with respect to electromyography (EMG) onset. The largest MEG signal peak was detected 8 ms prior to EMG onset. [3] Tongue SEFs obtained using pin electrodes (see Figure 2). The time window was set from −50 ms to 500 ms with respect to stimulus onset. The largest peak was detected 71 ms after stimulation onset. **(C)** ECD locations over the left hemisphere for the CMC and SEFs in one participant. The ECDs of the low-CMC and SEFs were positioned on the posterior bank of the central sulcus. The ECDs of the β-CMC were positioned on the anterior bank of the central sulcus. **(D)** The bar graph shows the mean amplitude of β-CMC for the tongue (mean ± standard error of the mean). Dashed lines represent the level of 99% statistical significance with the confidence limit. The β-CMC was significantly larger over the contralateral hemisphere than it was over the ipsilateral hemisphere for both sides of the tongue. *P < 0.05; Con, Contralateral hemisphere; Ipsi, Ipsilateral hemisphere. Images modified with permission from Maezawa et al. (2016a). Graph reproduced with permission from Maezawa et al. (2014b).

As previously mentioned, MRCFs have been detected over both hemispheres during self-paced tongue protrusion (“MRCFs Associated with Tongue Protrusions” Section), suggesting that both hemispheres contribute to tongue protrusion in humans. However, MRCF data are unable to reveal the pattern of dominance for functional connections between each side of the tongue and each hemisphere, as it is impossible to protrude the tongue forward using each side of the tongue separately due to the anatomical reasons, since the tongue is located along the midline of the human body. In contrast, it is easy to move fingers on each side of the hand separately. Research has suggested that CMC analyses are effective for the evaluation of functional connections between each hemisphere of the sensorimotor cortex and each side of the target peripheral muscles during sustained muscle contraction (Mima and Hallett, 1999). Recently, we detected significant, contralateral-dominant CMC in the β frequency band (β-CMC) over both hemispheres for each side of the tongue during isometric forward tongue protrusion (Figure 5; Maezawa et al., 2014b). These results suggest that the functional connection is stronger over the contralateral hemisphere for either side of the tongue during isometric tongue protrusion. These findings are in contrast with those for simultaneous isometric finger contractions on both sides, where β-CMC was only detected in the contralateral hemisphere for each side of the finger. Furthermore, the amplitude of β-CMC in the contralateral hemisphere was larger than that in the ipsilateral hemisphere for each side of the tongue. These findings are consistent with the results of transcranial magnetic stimulation studies, in which stimulation elicited motor-evoked potentials in both sides of the tongue. Such studies have also reported that the motor-evoked potential amplitudes of the contralateral side are greater than those of the ipsilateral side (Meyer et al., 1997; Ghezzi and Baldini, 1998; Rödel et al., 2003). These results may reflect contralateral dominance in the functional connections between each side of the tongue and each hemisphere during isometric tongue protrusion in humans.

**Figure 6:**
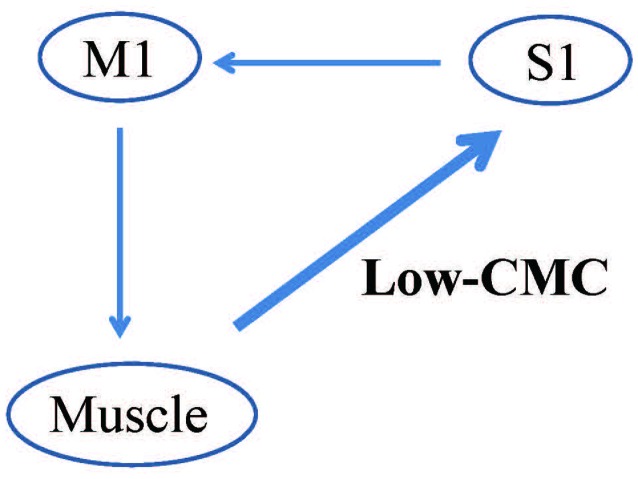
**Proposed pattern of CMC within the low-frequency band (low-CMC) during isometric tongue protrusion.** Low-CMC may mainly reflect proprioceptive feedback from the tongue muscles to the S1. This proposed feedback loop constitutes an M1-muscle-S1-M1 network, in contrast to the current hypothesis that CMC at the β band primarily reflects motor commands issued by the M1 to the tongue muscles. M1, primary motor cortex; S1, primary somatosensory cortex, Low-CMC, cortico-muscular coherence within the low-frequency band. Images modified with permission from Maezawa (2016).

Additionally, CMC within the low-frequency band (low-CMC; 2–10 Hz) was consistently detected in subjects of our previous studies (Figure 5; Maezawa et al., 2014b, 2016a), even though low-CMC was not consistently observed during finger contraction in earlier studies (Conway et al., 1995; Mima and Hallett, 1999). Time-domain analyses revealed that the MEG signal followed the EMG signal for low-CMC, but preceded the EMG signal for β-CMC. Moreover, the current sources of low-CMC were localized to the tongue region of S1, suggesting that low-CMC primarily reflects afferent feedback from the tongue to the cortex. However, this assumption is in direct contrast with the hypothesis that β-CMC mainly reflects efferent motor commands from the cortex to the tongue. Such bidirectional oscillatory information flow at two different frequency bands between the cortex and tongue may be critical for sophisticated tongue movement. Indeed, low-CMC may reflect activity within a sensorimotor feedback loop consisting of an M1-muscle-S1-M1 network (Figure 6; Maezawa, 2016).

In a recent MEG study by Ruspantini et al. (2012), CMC was detected between the sensorimotor cortex and orbicularis oris muscle in the low frequency band of speech rhythmicity (2–3 Hz) during speech production, suggesting that oscillatory activation related to proprioception from the oral regions may be important for executing orofacial movements during spoken language. Furthermore, partial directed coherence (PDC) analyses can be applied to determine the direction of cortico-muscular connectivity (Schelter et al., 2006; Faes and Nollo, 2010). An MEG study using PDC analysis of accelerometer data revealed that cortico-kinematic coherence at the low frequency band mainly reflects proprioceptive feedback from the muscle spindles to SM1 during repetitive finger movements (Bourguignon et al., 2015). Such findings support the notion that PDC analyses are useful for revealing the precise direction of information flow between the cortex and tongue for low-CMC.

## Clinical Applications

In addition to its research-based application, MEG is also useful for the evaluation of sensorimotor function in clinical settings. In such settings, MEG is most commonly used to record SEFs following median nerve stimulation, which aids in the assessment of abnormal S1 function in patients with cerebrovascular diseases (Maclin et al., 1994; Wikström et al., 1999), cortical reflex myoclonus (Uesaka et al., 1993, 1996; Karhu et al., 1994; Mima et al., 1998), and polymicrogyria (Ishitobi et al., 2005). In this section, we introduce the clinical utility of performing SEF recordings during tongue stimulation for the assessment sensory abnormalities of the tongue caused by lingual nerve damage.

**Figure 7:**
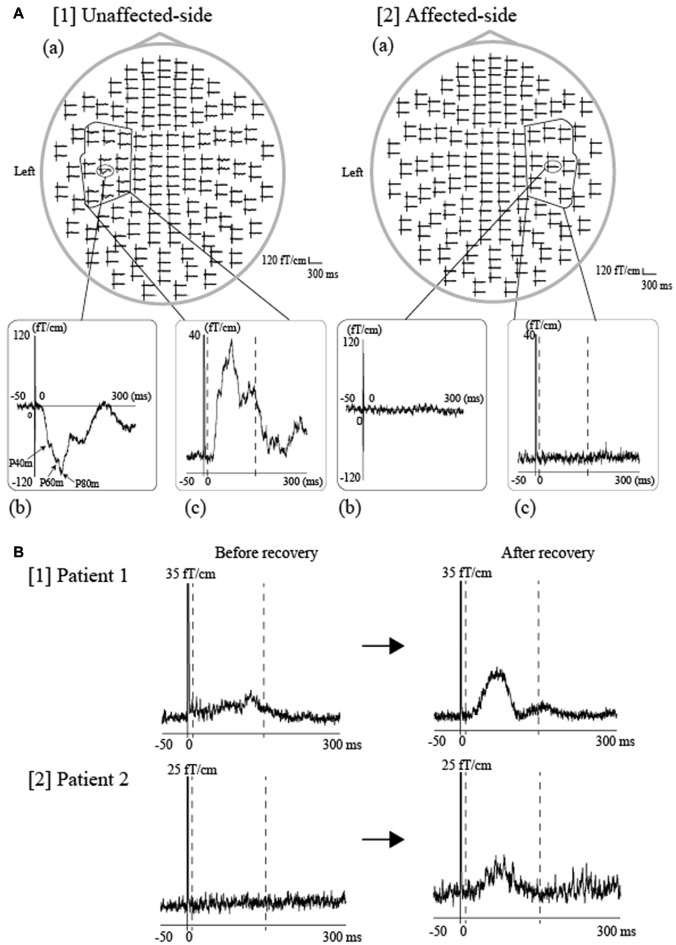
**SEFs elicited by tongue stimulation using pin electrodes in patients with tongue sensory disturbances. (A)** Tongue SEF waveforms in a patient with unilateral sensory disturbances of the tongue caused by oral surgery-induced lingual nerve injury. The same stimulation intensity was applied for both sides of the tongue at four times the sensory threshold in the unaffected side. The whole-head SEF waveforms following unaffected-side (right-side) ([1](a)) and affected-side (left-side) stimulation ([2](a)). The time window of each waveform was set from −50 ms to 300 ms with respect to stimulus onset. Obvious responses were observed over the contralateral hemisphere during unaffected-side (right-side) stimulation ([1] (b)), though no responses were detected during affected-side (left-side) stimulation ([2] (b)). To evaluate cortical activity using an objective parameter, we calculated the activated root-mean-square (aRMS) using spatial and temporal summation, which utilizes the time-averaged activity (between 10 and 150 ms) obtained from 18-channel RMS over the contralateral hemisphere (unaffected side: [1(C)], affected side: [2(C)]). The time points of 10 ms and 150 ms are indicated by the two dashed lines, respectively.** (B)** SEFs were measured prior to and following surgery in two patients who exhibited recovery from sensory impairments following oral surgery for repair of an injured lingual nerve. Subsequent evaluation revealed that sensory function improved by two score levels (British Medical Research Council [BMRC] criteria) in Patient 1 and by three levels in Patient 2. The 18-channel RMS waveforms evoked by stimulation of the affected side were observed before and after surgery in both patients (Patient 1, [1], Patient 2, [2]). The vertical scales are 35 fT/cm and 25 fT/cm, respectively. The SEF amplitude after the sensory recovery was obviously larger than that before sensory recovery in both patients. An objective parameter, referred to as the laterality index, which was calculated from the RMS amplitude between the affected and unaffected side (details described in our previous studies Maezawa et al., 2008, 2011), was out of the normal range in both patients before sensory recovery but was within the normal range after sensory recovery. The normal range was set to the average ±3 standard deviations of the laterality index from 10 healthy volunteers. Images modified with permission from Maezawa et al. (2016d).

Lingual nerve injury can occur during diverse interventions in the oral area, including third molar extraction, dental implant operation, root canal procedure, mandibular cyst removal, and local anesthetic injection. For example, the incidence of lingual injury during third molar extraction has been reported between 0.06%–10% (Kim et al., 2004). Indeed, sensory impairment of the tongue due to lingual nerve injury is a rare complication, although such injuries can sometimes result in speech disorders and eating difficulties, leading to a significant reduction in a patient’s quality of life. However, most clinicians employ subjective methods, including the von Frey test and two-point discrimination test, when assessing tongue sensory disturbances, while objective evaluation methods remain to established. Previously, we demonstrated that tongue SEFs have the potential to be an effective and objective parameter for detecting sensory abnormalities of the unilateral side of the tongue (Maezawa et al., 2011). Since intra-participant similarities in SEF waveforms have been detected between the right and left sides of the tongue in healthy volunteers (Maezawa et al., 2008), we utilized the unaffected side of the tongue as a control to evaluate tongue sensory disturbances. Such analysis allowed us to successfully estimate the degree of unilateral sensory disturbance in the tongue of each patient, using a laterality index for tongue SEFs between the affected and unaffected sides (Figure 7). Moreover, we recently reported two cases in which tongue SEFs were useful for evaluating sensory recovery, further supporting the notion that SEFs can be utilized for objective follow-up assessment (Maezawa et al., 2016d). Both patients recovered tongue sensation following surgery on the injured lingual nerve, as stimulation of the affected side—which had failed to evoke obvious cortical activity before surgery—exhibited clear cortical activity in both patients following surgery (Figure 7). Similarly, Yamashita et al. (1999) recorded SEFs during tactile tongue stimulation in three patients who underwent flap reconstruction for tongue carcinoma. The authors demonstrated that the patients recovered flap sensation following surgery, as SEFs were detected at a peak latency of between 20 ms and 40 ms following the edge of tongue stimulation both on the reconstructed side and on the unaffected side. These findings illustrate that SEFs provide objective evidence of tongue somatosensory impairments, and as such, are a useful method for evaluating tongue sensory disturbances in clinical situations.

In the future, it will be important to focus on movement disorders of the tongue, including dystonia and dyskinesia, as well as on tongue sensory abnormalities. For example, functional connectivity analyses using CMC may help to reveal the pathophysiological cortical mechanisms underlying tongue dystonia and dyskinesia. Obtaining CMC recordings during weak sustained protrusion of the tongue muscle may be particularly useful for patients with movement disorders, since this type of movement task is relatively easy for these patients, who may have difficulty performing repetitive, self-paced movement tasks during MRCFs measurement.

## Conclusions and Future Directions

The present review focused on the current understanding of tongue sensorimotor functions, as elucidated by MEG analyses over the past three decades. Research in this field is also being performed by combining MEG and EEG, or by using functional magnetic resonance imaging and positron emission tomography, which are advantageous owing to their high spatial resolutions (Shibasaki, 2008). Such multi-modal imaging studies may allow both researchers and clinicians to obtain a greater amount of spatiotemporal information.

Future studies should address the difficulty in obtaining MEG data for orofacial regions due to contamination artifacts induced by stimulation and/or muscle activity. For example, it is sometimes difficult to discriminate cortical responses from stimulus-induced artifacts when evaluating SEFs. To overcome this issue, Maezawa et al. (2008) delivered stimuli to the tongue with low intensity using pin electrodes, successfully reducing the number of stimulus-induced artifacts. Sakamoto et al. (2008a) recorded EMG signals from the anterior suprahyoid muscle to distinguish cortical activity from stimulation-induced artifacts related to tongue EMG activity. Additional studies have indicated that mechanical stimulation is also useful for stimulating the oral region, thereby eliminating the influence of electrical artifacts. Such methods include air puff stimulation using compressed nitrogen (Yoshida et al., 2006) and stimulation using a piezo-driven tactile stimulation device (Tamura et al., 2008). In addition, tongue movement tasks may induce activity in other facial muscles, such as the temporal and masseter muscles, during the measurement of MRCFs and CMC. In such cases, confirming the inactivity of the temporal and/or masseter muscles via simultaneous EMG recording during tongue movement tasks may be useful.

Recent developments in the field of brain-machine interfaces (BMI) have shown promise for the investigation of tongue motor functions in humans. BMI refers to a computer-based system that can decode motor commands from cortical signals and use them to control external devices to perform a desired action. The high temporal resolution of MEG has enabled some researchers to successfully predict the movement trajectories of the hand from cortical activity in real time (Georgopoulos et al., 2005), as well as the direction of small hand movements from single-trial recordings (Waldert et al., 2008). Other reports have utilized imaging techniques such as functional magnetic resonance imaging (Kamitani and Tong, 2006; Kay et al., 2008; Mitchell et al., 2008; Miyawaki et al., 2008), EEG (Birbaumer et al., 1999; Wolpaw and McFarland, 2004; Pfurtscheller and Neuper, 2006; Blankertz et al., 2007), and invasive-EEG (Leuthardt et al., 2004; Mehring et al., 2004) to predict human brain activity. Decoding the motor processing of the tongue region may be helpful for improving motor dysfunctions of the tongue in patients with dysphagia or speech movement disorders. For example, changes in the oscillatory power of the α and β bands in the sensorimotor cortex can be used to operate a BMI system, since ERD/ERS can be modulated by motor imagery as well as actual motor movements. Indeed, recent studies have revealed that ERD/ERS is induced by tongue stimulation, and that stimulus-induced ERD/ERS is modulated by tongue movement in most healthy participants (Maezawa et al., 2016c). Furthermore, this pattern of modulation is similar to that observed for upper limb regions (Pfurtscheller and Aranibar, 1977; Pfurtscheller and Lopes da Silva, 1999).

Another research area that has gained increased attention in recent years is the maturation of tongue sensorimotor systems from the neonatal to adolescent stage. In a previous study, Gaetz et al. (2010) recorded visually cued ERD/ERS associated with finger movement in both children (4–6 years old) and adolescents (11–13 years old). They then compared their findings with ERD/ERS data from adults, noting that the post-movement β-ERS in children was significantly decreased compared to that in adults and adolescents. The authors concluded that these findings may indicate that children exhibit decreased motor cortical inhibition relative to adolescents and adults. In addition, Cheyne et al. (2014) reported that the transition between immature and adult-like cortical responses associated with motor tasks occurs between 3–5 and 8 years old, as preschool children under 5 years old exhibited significantly different patterns of MRCFs associated with self-initiated index finger movement in comparison to older children and adults.

Some recent studies have also investigated aspects of somatosensory cortical development using MEG (Lauronen et al., 2012; Nevalainen et al., 2012, 2014) by recording stable SEFs following tactile stimulation of the finger in healthy newborns. The authors of these studies reported successful discrimination of cortical activity derived from S1 and S2, suggesting that the distinct patterns of somatosensory processing in both S1 and S2 in newborns relative to those of adults are a direct consequence of cortical immaturity. For example, activity from S2 was prominent during quiet sleep in neonates, in contrast to the diminished or absent S2 activity observed during non-REM sleep in adults. During the acquisition of oral functions, such as feeding and swallowing, it is important for children to preserve energy for growth. Therefore, studies examining developmental alterations in sensorimotor processing of the tongue may provide novel insight into the normal development of tongue sensorimotor processing. However, the limitations of MEG analyses in children (especially newborns) should be considered (Nevalainen et al., 2014). The most challenging of these problems to overcome is the inability to hold the child’s head in a stable position during MEG recording, as the sensor helmet is designed for use in adults. Furthermore, it is often difficult to instruct young children to hold their heads still during recording.

## Author Contributions

HM drafted, revised and approved final version of the manuscript.

## Funding

This work was supported by a Grant-in-Aid for Scientific Research (C)16K11621 (HM) from the Japan Society for the Promotion of Science.

## Conflict of Interest Statement

The author declares that the research was conducted in the absence of any commercial or financial relationships that could be construed as a potential conflict of interest.

## Abbreviations

BA: Brodmann area
BMI: brain-machine interfaces
BP: Bereitschaftspotential
CMC: cortico-muscular coherence
CN: cranial nerve
ECD: equivalent current dipole
EEG: electroencephalogram
EMG: electromyogram
ERD/ERS: event-related desynchronization/event-related synchronization
M1: primary motor cortex
MEF: motor-evoked field
MEG: magnetoencephalography
MF: motor field
MRCF: movement-related cortical field
MRCP: movement-related cortical potentials
RF: readiness field
S1: primary somatosensory cortex
S2: secondary somatosensory cortex
SEF: somatosensory evoked field
TSE: temporal spectral evolution.

## References

[B1] Birbaumer N. Ghanayim N. Hinterberger T. Iversen I. Kotchoubey B. Kübler A.. (1999). A spelling device for the paralysed. Nature 398, 297–298. 10.1038/18581 10192330

[B2] Blankertz B. Dornhege G. Krauledat M. Müller K. R. Curio G. (2007). The non-invasive Berlin brain-computer interface: fast acquisition of effective performance in untrained subjects. Neuroimage 37, 539–550. 10.1016/j.neuroimage.2007.01.051 17475513

[B3] Bourguignon M. Piitulainen H. De Tiège X. Jousmäki V. Hari R. (2015). Corticokinematic coherence mainly reflects movement-induced proprioceptive feedback. Neuroimage 106, 382–390. 10.1016/j.neuroimage.2014.11.026 25463469 PMC4295920

[B4] Brenner D. Lipton J. Kaufman L. Williamson S. J. (1978). Somatically evoked fields of the human brain. Science 199, 81–83. 10.1126/science.199.4324.81 17569490

[B5] Caetano G. Jousmäki V. Hari R. (2007). Actor’s and observer’s primary motor cortices stabilize similarly after seen or heard motor actions. Proc. Natl. Acad. Sci. U S A 104, 9058–9062. 10.1073/pnas.0702453104 17470782 PMC1859994

[B6] Chatrian G. E. Petersen M. C. Lazarte J. A. (1959). The blocking of the rolandic wicket rhythm and some central changes related to movement. Electroencephalogr. Clin. Neurophysiol. 11, 497–510. 10.1016/0013-4694(59)90048-3 13663823

[B9] Cheyne D. O. (2013). MEG studies of sensorimotor rhythms: a review. Exp. Neurol. 245, 27–39. 10.1016/j.expneurol.2012.08.030 22981841

[B7] Cheyne D. Jobst C. Tesan G. Crain S. Johnson B. (2014). Movement-related neuromagnetic fields in preschool age children. Hum. Brain Mapp. 35, 4858–4875. 10.1002/hbm.22518 24700413 PMC6869527

[B8] Cheyne D. Kristeva R. Deecke L. (1991). Homuncular organization of human motor cortex as indicated by neuromagnetic recordings. Neurosci. Lett. 122, 17–20. 10.1016/0304-3940(91)90182-s 2057131

[B10] Conway B. Halliday D. Farmer S. F. Shahani U. Maas P. Weir A. I.. (1995). Synchronization between motor cortex and spinal motoneuronal pool during the performance of a maintained motor task in man. J. Physiol. 489, 917–924. 10.1113/jphysiol.1995.sp021104 8788955 PMC1156860

[B11] Crone N. E. Miglioretti D. L. Gordon B. Sieracki J. M. Wilson M. T. Uematsu S.. (1998). Functional mapping of human sensorimotor cortex with electrocorticographic spectral analysis. I. Alpha and beta event-related desynchronization. Brain 121, 2271–2299. 10.1093/brain/121.12.2271 9874480

[B12] Deecke L. Engel M. Lang W. Komhuber H. H. (1986). Bereitschaftspotential preceding speech after breath holding. Exp. Brain Res. 65, 219–223. 10.1007/bf00243845 3803506

[B13] Deecke L. Weinberg H. Brickett P. (1982). Magnetic fields of the human brain accompanying voluntary movement: bereitschaftsmagnetfeld. Exp. Brain Res. 48, 144–148. 10.1007/bf00239582 7140885

[B14] Disbrow E. A. Hinkley L. B. N. Roberts T. P. L. (2003). Ipsilateral representation of oral structures in human anterior parietal somatosensory cortex and integration of inputs across the midline. J. Comp. Neurol. 467, 487–495. 10.1002/cne.10935 14624483

[B15] Dziewas R. Sörös P. Ishii R. Chau W. Henningsen H. Ringelstein E. B.. (2003). Neuroimaging evidence for cortical involvement in the preparation and in the act of swallowing. Neuroimage 20, 135–144. 10.1016/s1053-8119(03)00285-4 14527576

[B16] Engel A. Fries P. (2010). Beta-band oscillations—signaling the status quo? Curr. Opin. Neurobiol. 20, 156–165. 10.1016/j.conb.2010.02.015 20359884

[B17] Faes L. Nollo G. (2010). Extended causal modeling to assess Partial Directed Coherence in multiple time series with significant instantaneous interactions. Biol. Cybern. 103, 387–400. 10.1007/s00422-010-0406-6 20938676

[B18] Furlong P. L. Hobson A. R. Aziz Q. Barnes G. R. Singh K. D. Hillebrand A.. (2004). Dissociating the spatio-temporalcharacteristics of cortical neuronal activity associated with human volitional swallowing in the healthy adult brain. Neuroimage 22, 1447–1455. 10.1016/j.neuroimage.2004.02.041 15275902

[B19] Gaetz W. Macdonald M. Cheyne D. Snead O. C. (2010). Neuromagnetic imaging of movement-related cortical oscillations in children and adults: age predicts post-movement beta rebound. Neuroimage 51, 792–807. 10.1016/j.neuroimage.2010.01.077 20116434

[B20] Gastaut H. Terzian H. Gastaut Y. (1952). Study of a little electroencephalographic activity: rolandic arched rhythm. Mars. Med. 89, 296–310. 12991978

[B21] Georgopoulos A. P. Langheim F. J. P. Leuthold A. C. Merkle A. N. (2005). Magnetoencephalographic signals predict movement trajectory in space. Exp. Brain Res. 167, 132–135. 10.1007/s00221-005-0028-8 16044305

[B22] Ghezzi A. Baldini S. (1998). A simple method for recording motor evoked potentials of lingual muscles to transcranial magnetic and peripheral electrical stimulation. Electroencephalogr. Clin. Neurophysiol. 109, 114–118. 10.1016/s0924-980x(98)00008-3 9741801

[B23] Gilbertson T. Lalo E. Doyle L. Di Lazzaro V. Cioni B. Brown P. (2005). Existing motor state is favored at the expense of new movement during 13–35 Hz oscillatory synchrony in the human corticospinal system. J. Neurosci. 25, 7771–7779. 10.1523/jneurosci.1762-05.2005 16120778 PMC6725263

[B25] Gunji A. Ishii R. Chau W. Kakigi R. Pantev C. (2007). Rhythmic brain activities related to singing in humans. Neuroimage 34, 426–434. 10.1016/j.neuroimage.2006.07.018 17049276

[B26] Gunji A. Kakigi R. Hoshiyama M. (2000). Spatiotemporal source analysis of vocalization-associated magnetic fields. Cogn. Brain Res. 9, 157–163. 10.1016/s0926-6410(99)00054-3 10729699

[B27] Hamdy S. Mikulis D. Crawley A. Xue S. Lau H. Henry S.. (1999a). Cortical activation during human volitional swallowing: an event-related fMRI study. Am. J. Physiol. 277, 219–225. 10409170 10.1152/ajpgi.1999.277.1.G219

[B28] Hamdy S. Rothwell J. Brooks D. Bailey D. Aziz Q. Thompson D. (1999b). Identification of the cerebral loci processing human swallowing with H_2_^15^O PET activation. J. Neurophysiol. 81, 1917–1926. 10200226 10.1152/jn.1999.81.4.1917

[B32] Hari R. Antervo A. Katila T. Poutanen T. Seppänen M. Tuomisto T. (1983a). Cerebral magnetic fields associated with voluntary movements in man. Nuovo. Cimento 2, 484–494. 10.1007/bf02455947

[B33] Hari R. Kaukoranta E. Reinikainen K. Huopaniemie T. Mauno J. (1983b). Neuromagnetic localization of cortical activity evoked by painful dental stimulation in man. Neurosci. Lett. 42, 77–82. 10.1016/0304-3940(83)90425-1 6657149

[B29] Hari R. Salenius S. (1999). Rhythmical corticomotor communication. Neuroreport 10, R1–R10. 10203308

[B30] Hari R. Salmelin R. (1997). Human cortical oscillations: a neuromagnetic view through the skull. Trends Neurosci. 20, 44–49. 10.1016/s0166-2236(96)10065-5 9004419

[B31] Hari R. Salmelin R. (2012). Magnetoencephalography: from SQUIDs to neuroscience. Neuroimage 20th anniversary special edition. Neuroimage 61, 386–396. 10.1016/j.neuroimage.2011.11.074 22166794

[B34] Hillebrand A. Barnes G. R. (2002). A quantitative assessment of the sensitivity of whole-head MEG to activity in the adult human cortex. Neuroimage 16, 638–650. 10.1006/nimg.2002.1102 12169249

[B35] Huckabee M. L. Deecke L. Cannito M. P. Gould H. J. Mayr W. (2003). Cortical control mechanisms in volitional swallowing: the bereitschaftspotential. Brain Topogr. 16, 3–17. 10.1023/A:1025671914949 14587965

[B36] Ikeda A. Lüders H. O. Burgess R. C. Sakamoto A. Klem G. H. Morris H. H. III. (1995). Generator locations of movement-related potentials with tongue protrusions and vocalizations: subdural recording in human. Electroencephalogr. Clin. Neurophysiol. 96, 310–328. 10.1016/0168-5597(95)00002-a 7635076

[B37] Ishitobi M. Nakasato N. Yoshimoto T. Iinuma K. (2005). Abnormal primary somatosensory function in unilateral polymicrogyria: an MEG study. Brain Dev. 27, 22–29. 10.1016/j.braindev.2004.02.013 15626537

[B38] Jain N. Qi H.-X. Catania K. Kaas J. (2001). Anatomic correlates of the face and oral cavity representations in the somatosensory cortical area 3b of monkeys. J. Comp. Neurol. 429, 455–468. 10.1002/1096-9861(20010115)429:3<455::AID-CNE7>3.0.CO;2-F 11116231

[B40] Jasper H. H. Andrews H. L. (1938). Electro-encephalography III. Normal differentiation of occipital and precentral regions in man. Arch. Neurol. Psychiatry 39, 96–115. 10.1001/archneurpsyc.1938.02270010106010

[B39] Jasper H. Penfield W. (1949). Electrocorticograms in man: effect of voluntary movement upon the electrical activity of the precentral gyrus. Arch. Psychiatr. Nervenkr. 183, 163–174. 10.1007/bf01062488

[B41] Jean A. (1984). Brainstem organization of the swallowing network. Brain Behav. Evol. 25, 109–116. 10.1159/000118856 6100081

[B42] Joundi R. A. Jenkinson N. Brittain J. S. Aziz T. Z. Brown P. (2012). Driving oscillatory activity in the human cortex enhances motor performance. Curr. Biol. 22, 403–407. 10.1016/j.cub.2012.01.024 22305755 PMC3343257

[B43] Kakigi R. Hoshiyama M. Shimojo M. Naka D. Yamasaki H. Watanabe S.. (2000). The somatosensory evoked magnetic fields. Prog. Neurobiol. 61, 495–523. 10.1016/S0301-0082(99)00063-5 10748321

[B44] Kamitani Y. Tong F. (2006). Decoding seen and attended motion directions from activity in the human visual cortex. Curr. Biol. 16, 1096–1102. 10.1016/j.cub.2006.04.003 16753563 PMC1635016

[B45] Karhu J. Hari R. Lu S.-T. Paetau R. Rif J. (1991). Cerebral magnetic fields to lingual stimulation. Electroencephalogr. Clin. Neurophysiol. 80, 459–468. 10.1016/0168-5597(91)90127-j 1720721

[B46] Karhu J. Hari R. Paetau R. Kajola M. Mervaala E. (1994). Cortical reactivity in progressive myoclonus epilepsy. Electroencephalogr. Clin. Neurophysiol. 90, 93–102. 10.1016/0013-4694(94)90001-9 7510633

[B47] Kaufman L. Okada Y. Brenner D. Williamson S. J. (1981). On the relation between somatic evoked potentials and fields. Int. J. Neurosci. 15, 223–239. 10.3109/00207458108985860 7319709

[B48] Kay K. N. Naselaris T. Prenger R. J. Gallant J. (2008). Identifying natural images from human brain activity. Nature 452, 352–355. 10.1038/nature06713 18322462 PMC3556484

[B49] Kim S. Y. Hu K. S. Chung I. H. Lee E. W. Kim H. J. (2004). Topographic anatomy of the lingual nerve and variations in communication pattern of the mandibular nerve branches. Surg. Radiol. Anat. 26, 128–135. 10.1007/s00276-003-0179-x 14586562

[B50] Lauronen L. Nevalainen P. Pihko E. (2012). Magnetoencephalography in neonatology. Neurophysiol. Clin. 42, 27–34. 10.1016/j.neucli.2011.08.006 22200339

[B51] Leuthardt E. C. Schalk G. Wolpaw J. R. Ojemann J. G. Moran D. W. (2004). A brain-computer interface using electrocorticographic signals in humans. J. Neural Eng. 1, 63–71. 10.1088/1741-2560/1/2/001 15876624

[B52] Little S. Brown P. (2014). The functional role of beta oscillations in Parkinson’s disease. Parkinsonism Relat. Disord. 20, S44–S48. 10.1016/s1353-8020(13)70013-0 24262186

[B53] Maclin E. L. Rose D. F. Knight J. E. Orrison W. W. Davis L. E. (1994). Somatosensory evoked magnetic fields in patients with stroke. Electroencephalogr. Clin. Neurophysiol. 91, 468–475. 10.1016/0013-4694(94)90167-8 7529685

[B54] Maezawa H. (2016). Cortico-muscular communication for motor control of the tongue in humans: a review. J. Oral Biosci. 58, 69–72. 10.1016/j.job.2016.03.001

[B55] Maezawa H. Hirai Y. Shiraishi H. Funahashi M. (2014a). Somatosensory evoked magnetic fields following tongue and hard palate stimulation on the preferred chewing side. J. Neurol. Sci. 347, 288–294. 10.1016/j.jns.2014.10.025 25455302

[B56] Maezawa H. Mima T. Yazawa S. Matsuhashi M. Shiraishi H. Hirai Y.. (2014b). Contralateral dominance of corticomuscular coherence for both sides of the tongue during human tongue protrusion: an MEG study. Neuroimage 101, 245–255. 10.1016/j.neuroimage.2014.07.018 25038437

[B57] Maezawa H. Mima T. Yazawa S. Matsuhashi M. Shiraishi H. Funahashi M. (2016a). Cortico-muscular synchronization by proprioceptive afferents from the tongue muscles during isometric tongue protrusion. Neuroimage 128, 284–292. 10.1016/j.neuroimage.2015.12.058 26774611

[B58] Maezawa H. Oguma H. Hirai Y. Hisadome K. Shiraishi H. Funahashi M. (2016b). Movement-related cortical magnetic fields associated with self-paced tongue protrusion in humans. Neurosci. Res. [Epub ahead of print]. 10.1016/j.neures.2016.11.010 27888072

[B59] Maezawa H. Onishi K. Yagyu K. Shiraishi H. Hirai Y. Funahashi M. (2016c). Modulation of stimulus-induced 20-Hz activity for the tongue and hard palate during tongue movement in humans. Clin. Neurophysiol. 127, 698–705. 10.1016/j.clinph.2015.06.007 26116299

[B60] Maezawa H. Tojyo I. Yoshida K. Fujita S. (2016d). Recovery of impaired somatosensory evoked fields after improvement of tongue sensory deficits with neurosurgical reconstruction. J. Oral Maxillofac. Surg. 74, 1473–1482. 10.1016/j.joms.2016.01.011 26855025

[B61] Maezawa H. Yoshida K. Matsuhashi M. Yokoyama Y. Mima T. Bessho K.. (2011). Evaluation of tongue sensory disturbance by somatosensory evoked magnetic fields following tongue stimulation. Neurosci. Res. 71, 244–250. 10.1016/j.neures.2011.07.1831 21821071

[B62] Maezawa H. Yoshida K. Nagamine T. Matsubayashi J. Enatsu R. Bessho K.. (2008). Somatosensory evoked magnetic fields following electric tongue stimulation using pin electrodes. Neurosci. Res. 62, 131–139. 10.1016/j.neures.2008.07.004 18708103

[B63] Manger P. R. Woods T. M. Jones E. G. (1995). Representation of the face and intraoral structures in area 3b of the squirrel monkey (*Saimiri sciureus*) somatosensory cortex, with special reference to the ipsilateral representation. J. Comp. Neurol. 362, 597–607. 10.1002/cne.903620412 8636470

[B64] Manger P. R. Woods T. M. Jones E. G. (1996). Representation of face and intra-oral structures in area 3b of macaque monkey somatosensory cortex. J. Comp. Neurol. 371, 513–521. 10.1002/(SICI)1096-9861(19960805)371:4<513::AID-CNE2>3.0.CO;2-7 8841906

[B66] Martin R. E. Murray G. M. Kemppainen P. Masuda Y. Sessle B. J. (1997). Functional properties of neurons in the primate tongue primary motor cortex during swallowing. J. Neuophysiol. 78, 1516–1530. 9310440 10.1152/jn.1997.78.3.1516

[B65] Martin R. Sessle B. (1993). The role of the cerebral cortex in swallowing. Dysphagia 8, 195–202. 10.1007/bf01354538 8359039

[B67] Martin-Harris B. (2006). Coordination of respiration and swallowing. GI Motility Online. 10.1038/gimo10

[B68] Matsuo K. Palmer J. B. (2009). Coordination of mastication, swallowing and breathing. Jpn. Dent. Sci. Rev. 45, 31–40. 10.1016/j.jdsr.2009.03.004 20161022 PMC2749282

[B69] McAdam D. W. Whitaker H. A. (1971). Electroencephalographic localization in the normal human brain. Science 172, 499–502. 10.1126/science.172.3982.499 5550508

[B70] Mehring C. Nawrot M. P. de Oliveira S. C. Vaadia E. Schulze-Bonhage A. Aertsen A.. (2004). Comparing information about arm movement direction in single channels of local and epicortical field potentials from monkey and human motor cortex. J. Physiol. Paris. 98, 498–506. 10.1016/j.jphysparis.2005.09.016 16310349

[B71] Meyer B. U. Liebsch R. Rörich S. (1997). Tongue motor responses following transcranial magnetic stimulation of the motor cortex and proximal hypoglossal nerve in man. Electroencephalogr. Clin. Neurophysiol. 105, 15–23. 10.1016/s0924-980x(96)96598-4 9118834

[B72] Miller K. J. Leuthardt E. C. Schalk G. Rao R. P. Anderson N. R. Moran D. W.. (2007). Spectral changes in cortical surface potentials during motor movement. J. Neurosci. 27, 2424–2432. 10.1523/JNEUROSCI.3886-06.2007 17329441 PMC6673496

[B73] Mima T. Hallett M. (1999). Corticomuscular coherence: a review. J. Clin. Neurophysiol. 16, 501–511. 10.1097/00004691-199911000-00002 10600018

[B74] Mima T. Nagamine T. Ikeda A. Yazawa S. Kimura J. Shibasaki H. (1998). Pathogenesis of cortical myoclonus studied by magnetoencephalography. Ann. Neurol. 43, 598–607. 10.1002/ana.410430507 9585353

[B75] Mitchell T. M. Shinkareva S. V. Carlson A. Chang K. M. Malave V. L. Mason R. A.. (2008). Predicting human brain activity associated with the meanings of nouns. Science 320, 1191–1195. 10.1126/science.1152876 18511683

[B76] Miyawaki Y. Uchida H. Yamashita O. Sato M. A. Morito Y. Tanabe H. C.. (2008). Visual image reconstruction fromhuman brain activity using a combination of multiscale local image decoders. Neuron 60, 915–929. 10.1016/j.neuron.2008.11.004 19081384

[B77] Mu L. Sanders I. (2010). Human tongue neuroanatomy: nerve supply and motor endplates. Clin. Anat. 23, 777–791. 10.1002/ca.21011 20607833 PMC2955167

[B78] Nagamine T. Kajola M. Salmelin R. Shibasaki H. Hari R. (1996). Movement-related slow cortical magnetic fields and changes of spontaneous MEG- and EEG-brain rhythms. Electroencephalogr. Clin. Neurophysiol. 99, 274–286. 10.1016/s0921-884x(96)95154-1 8862117

[B79] Nakahara H. Nakasato N. Kanno A. Murayama S. Hatanaka K. Itoh H.. (2004). Somatosensory-evoked fields for gingiva, lip and tongue. J. Dent. Res. 83, 307–311. 10.1177/154405910408300407 15044504

[B80] Nakamura A. Yamada T. Goto A. Kato T. Ito K. Abe Y.. (1998). Somatosensory homunculus as drawn by MEG. Neuroimage 7, 377–386. 10.1006/nimg.1998.0332 9626677

[B81] Nakasato N. Itoh H. Hatanaka K. Nakahara H. Kanno A. Yoshimoto T. (2001). Movement-related magnetic fields to tongue protrusion. Neuroimage 14, 924–935. 10.1006/nimg.2001.0881 11554811

[B82] Nevalainen P. Lauronen L. Pihko E. (2014). Development of human somatosensory cortical functions—what have we learned from magnetoencephalography: a review. Front. Hum. Neurosci. 8:158. 10.3389/fnhum.2014.00158 24672468 PMC3955943

[B83] Nevalainen P. Pihko E. Metsäranta M. Sambeth A. Wikström H. Okada Y.. (2012). Evoked magnetic fields from primary and secondary somatosensory cortices: a reliable tool for assessment of cortical processing in the neonatal period. Clin. Neurophysiol. 123, 2377–2383. 10.1016/j.clinph.2012.05.021 22749463

[B84] Ogawa H. Ito S. I. Nomura T. (1989). Oral cavity representation at the frontal operculum of macaque monkeys. Neurosci. Res. 6, 283–298. 10.1016/0168-0102(89)90021-7 2725988

[B85] Penfield W. Rasmussen T. (1950). The Cerebral Cortex of Man: A Clinical Study of Localization of Function. New York, NY: Macmillan.

[B86] Pfurtscheller G. Aranibar A. (1977). Event-related cortical desynchronization detected by power measurements of scalp EEG. Electroencephalogr. Clin. Neurophysiol. 42, 817–826. 10.1016/0013-4694(77)90235-8 67933

[B87] Pfurtscheller G. Lopes da Silva F. H. (1999). Event-related EEG/MEG synchronization and desynchronization: basic principles. Clin. Neurophysiol. 110, 1842–1857. 10.1016/s1388-2457(99)00141-8 10576479

[B88] Pfurtscheller G. Neuper C. (2006). Future prospects of ERD/ERS in the context of brain-computer interface (BCI) developments. Prog. Brain Res. 159, 433–437. 10.1016/s0079-6123(06)59028-4 17071247

[B89] Pogosyan A. Gaynor L. D. Eusebio A. Brown P. (2009). Boosting cortical activity at Beta-band frequencies slows movement in humans. Curr. Biol. 19, 1637–1641. 10.1016/j.cub.2009.07.074 19800236 PMC2791174

[B90] Rektor I. Sochurková D. Bocková M. (2006). Intracerebral ERD/ERS in voluntary movement and in cognitive visuomotor task. Prog. Brain Res. 159, 311–330. 10.1016/s0079-6123(06)59021-1 17071240

[B91] Rödel R. M. Laskawi R. Markus H. (2003). Tongue representation in the lateral cortical motor region of the human brain as assessed by transcranial magnetic stimulation. Ann. Otol. Rhinol. Laryngol. 112, 71–76. 10.1177/000348940311200114 12537062

[B92] Ruspantini I. Saarinen T. Belardinelli P. Jalava A. Parviainen T. Kujala J.. (2012). Corticomuscular coherence is tuned to the spontaneous rhythmicity of speech at 2-3 Hz. J. Neurosci. 32, 3786–3790. 10.1523/JNEUROSCI.3191-11.2012 22423099 PMC6703459

[B93] Sakamoto K. Nakata H. Kakigi R. (2008a). Somatosensory-evoked magnetic fields following stimulation of the tongue in humans. Clin. Neurophysiol. 119, 1664–1673. 10.1016/j.clinph.2008.03.029 18474450

[B94] Sakamoto K. Nakata H. Kakigi R. (2008b). Somatotopic representation of the tongue in human secondary somatosensory cortex. Clin. Neurophysiol. 119, 2125–2134. 10.1016/j.clinph.2008.05.003 18620905

[B95] Sakamoto K. Nakata H. Yumoto M. Kakigi R. (2010). Somatosensory processing of the tongue in humans. Front. Physiol. 1:136. 10.3389/fphys.2010.00136 21423377 PMC3059928

[B96] Salenius S. Schnitzler A. Salmelin R. Jousmäki V. Hari R. (1997). Modulation of human cortical rolandic rhythms during natural sensorimotor tasks. Neuroimage 5, 221–228. 10.1006/nimg.1997.0261 9345551

[B97] Salmelin R. (2007). Clinical neurophysiology of language: the MEG approach. Clin. Neurophysiol. 118, 237–254. 10.1016/j.clinph.2006.07.316 17008126

[B98] Salmelin R. Hari R. (1994a). Spatiotemporal characteristics of sensorimotor neuromagnetic rhythms related to thumb movement. Neuroscience 60, 537–550. 10.1016/0306-4522(94)90263-1 8072694

[B99] Salmelin R. Hari R. (1994b). Characterization of spontaneous MEG rhythms in healthy adults. Electroencephalogr. Clin. Neurophysiol. 91, 237–248. 10.1016/0013-4694(94)90187-2 7523073

[B100] Salmelin R. Sams M. (2002). Motor cortex involvement during verbal versus non-verbal lip and tongue movements. Hum. Brain Mapp. 16, 81–91. 10.1002/hbm.10031 11954058 PMC6871890

[B101] Satow T. Ikeda A. Yamamoto J. Begum T. Thuy D. H. Matsuhashi M.. (2004). Role of primary sensorimotor cortex and supplementary motor area in volitional swallowing: a movement-related cortical potential study. Am. J. Physiol. Gastrointest. Liver Physiol. 287, 459–470. 10.1152/ajpgi.00323.2003 14701719

[B102] Sawczuk A. Mosier K. M. (2001). Neural control of tongue movement with respect to respiration and swallowing. Crit. Rev. Oral. Biol. Med. 12, 18–37. 10.1177/10454411010120010101 11349959

[B103] Schelter B. Winterhalder M. Eichler M. Peifer M. Hellwig B. Guschlbauer B.. (2006). Testing for directed influences among neural signals using partial directed coherence. J. Neurosci. Methods 152, 210–219. 10.1016/j.jneumeth.2005.09.001 16269188

[B104] Shibasaki H. (2008). Human brain mapping: hemodynamic response and electrophysiology. Clin. Neurophysiol. 119, 731–743. 10.1016/j.clinph.2007.10.026 18187361

[B105] Shibasaki H. (2012). Cortical activities associated with voluntary movements and involuntary movements. Clin. Neurophysiol. 123, 229–243. 10.1016/j.clinph.2011.07.042 21906995

[B106] Shibasaki H. Hallett M. (2006). What is the Bereitschaftspotential? Clin. Neurophysiol. 17, 2341–2356. 10.1016/j.clinph.2006.04.025 16876476

[B107] Tamura Y. Shibukawa Y. Shintani M. Kaneko Y. Ichinohe T. (2008). Oral structure representation in human somatosensory cortex. Neuroimage 43, 128–135. 10.1016/j.neuroimage.2008.06.040 18672075

[B108] Uesaka Y. Terao Y. Ugawa Y. Yumoto M. Hanajima R. Kanazawa I. (1996). Magnetoencephalographic analysis of cortical myoclonic jerks. Electroencephalogr. Clin. Neurophysiol. 99, 141–148. 10.1016/0013-4694(96)95209-8 8761050

[B109] Uesaka Y. Ugawa Y. Yumoto M. Sakuta M. Kanazawa I. (1993). Giant somatosensory evoked magnetic field in patients with myoclonus epilepsy. Electroencephalogr. Clin. Neurophysiol. 87, 300–305. 10.1016/0013-4694(93)90183-v 7693441

[B110] van Wijk B. C. Beek P. J. Daffertshofer A. (2012). Neural synchrony within the motor system: what have we learned so far? Front. Hum. Neurosci. 6:252. 10.3389/fnhum.2012.00252 22969718 PMC3432872

[B111] Waldert S. Preissl H. Demandt E. Braun C. Birbaumer N. Aertsen A.. (2008). Hand movement direction decoded from MEG and EEG. J. Neurosci. 28, 1000–1008. 10.1523/JNEUROSCI.5171-07.2008 18216207 PMC6671004

[B112] Weinberg H. Brickett P. Deecke L. Boschert J. (1983). Slow magnetic fields of the brain preceding movement and speech. Il. Nuovo. Cimento. D 2, 495–504. 10.1007/bf02455948

[B113] Wikström H. Roine R. O. Salonen O. Lund K. B. Salli E. Ilmoniemi R. J.. (1999). Somatosensory evoked magnetic fields from the primary somatosensory cortex (SI) in acute stroke. Clin. Neurophysiol. 110, 916–923. 10.1016/s1388-2457(99)00026-7 10400206

[B114] Wohlert A. B. (1993). Event-related brain potentials preceding speech and nonspeech oral movements of varying complexity. J. Speech. Hear. Res. 36, 897–905. 10.1044/jshr.3605.897 8246478

[B115] Wolpaw J. R. McFarland D. J. (2004). Control of a two-dimensional movement signal by a noninvasive brain-computer interface in humans. Proc. Natl. Acad. Sci. U S A 101, 17849–17854. 10.1073/pnas.0403504101 15585584 PMC535103

[B116] Yamashita H. Kumamoto Y. Nakashima T. Yamamoto T. Inokuchi A. Komiyama S. (1999). Magnetic sensory cortical responses evoked by tactile stimulations of the human face, oral cavity and flap reconstructions of the tongue. Eur. Arch. Otorhinolaryngol. 256, 42–46. 10.1007/pl00014152 10337526

[B117] Yoshida K. Maezawa H. Nagamine T. Fukuyama H. Murakami K. Iizuka T. (2006). Somatosensory evoked magnetic fields to air-puff stimulation on the soft palate. Neurosci. Res. 55, 116–122. 10.1016/j.neures.2006.02.007 16677731

[B118] Zur K. B. Mu L. Sanders I. (2004). Distribution pattern of the human lingual nerve. Clin. Anat. 17, 88–92. 10.1002/ca.10166 14974094

